# Cavity-enhanced continuous-wave microscopy with potentially unstable cavity length

**DOI:** 10.1038/s41598-025-13589-w

**Published:** 2025-07-29

**Authors:** Oliver Lueghamer, Stefan Nimmrichter, Clara Conrad-Billroth, Thomas Juffmann, Maximilian Prüfer

**Affiliations:** 1 Vienna Center for Quantum Science and Technology, Atominstitut, TU Wien, Stadionallee 2, 1020 Vienna, Austria; 2 Naturwissenschaftlich-Technische Fakultät, Universität Siegen, 57068 Siegen, Germany; 3 Faculty of Physics, VCQ, University of Vienna, A-1090 Vienna, Austria; 4 Max Perutz Laboratories, Department of Structural and Computational Biology, University of Vienna, 1030 Vienna, Austria

**Keywords:** Microscopy, Ultracold gases

## Abstract

Microscopy gives access to spatially resolved dynamics in different systems, from biological cells to cold atoms. A big challenge is maximizing the information per used probe particle to limit the damage to the probed system. We present a cavity-enhanced continuous-wave microscopy approach that provides enhanced signal-to-noise ratios at fixed damage compared to standard single-pass microscopy. Employing a self-imaging 4f cavity, we show contrast enhancement for controlled test samples as well as biological samples. For thick samples, the imaging cavity leads to a new form of dark-field microscopy, where the separation of scattered and unscattered light is based on optical path length. We theoretically show that enhanced signal, signal-to-noise, and signal-to-noise per damage are also retrieved when the cavity is not length-stabilized. Our results provide an approach to cavity-enhanced microscopy with non-length-stabilized cavities and might be used to enhance the performance of dispersive imaging of ultracold atoms.

**Supplementary Information:**

The online version contains supplementary material available at 10.1038/s41598-025-13589-w.

Advances in microscopy have led to new discoveries across scientific disciplines^1^, from cellular biology^2^ to quantum physics^3^ and beyond. Microscopy setups measure certain parameters of interest in a spatially resolved way. Not only the spatial resolution limits the measurement precision but often the limiting factor is the finite number of probe particles detected in a given image. In many applications, the number of probe particles cannot be increased arbitrarily, either due to sample, source, or detector restrictions. Maximizing the information obtained from each detected probe particle is crucial in such applications.

For coherent imaging techniques, it has recently been shown that multi-passing each probe particle through the sample can increase the information per detected probe particle. This has been experimentally demonstrated in optical imaging and diffraction studies^4^, where self-imaging cavities^5^ were used to multi-pass a pulsed probe through a sample. Follow-up experiments showed the build-up of orbital angular momentum in a multi-passing experiment^7^. Notably, multi-passing probe particles *m* times through a sample can enable a measurement precision per probe-sample interaction similar to a quantum-enhanced measurement with *m* suitably entangled probe particles. It scales $\propto m$ instead of $\propto \sqrt{m}$^8–12^.

In physics applications, like spectroscopy or the imaging of ultracold atoms, a narrow spectral linewidth is often required. Thus, cavity-enhanced microscopy has to be done with continuous-wave (CW) excitation, which actually has theoretically been shown to outperform the pulsed multi-pass scheme^12^. However, despite the widespread use of single-mode cavity-enhanced measurements in various scientific and technological fields, applications in imaging remain rare, due to challenges in operating a cavity that is fully degenerate in all transverse modes^13^. The first progress was recently demonstrated in a 4-pass geometry that showed contrast enhancement in flow-cytommetry^14^. So far, genuine continuous-wave imaging cavities have been employed in the realization of multi-mode lasers^15^, multi-mode coherent absorbers^16^, cavity-enhanced non-linear optics applications^17^, and imaging of ultracold atoms^18^.

In this paper, we demonstrate cavity-enhanced continuous-wave microscopy using a self-imaging 4*f* cavity. We demonstrate the concept experimentally by applying it to the imaging of an artificially fabricated sample, consisting of holes in a $10\,\text {nm}$ thin silicon nitride ($\text {Si}_3\text {N}_4$) membrane. Imaging human cheek cells, we further demonstrate that cavity-based imaging leads to a novel form of dark-field microscopy, where the separation of scattered and unscattered light is based on optical path length and not on scattering angle. Our experiments are backed by theory which shows that an enhanced contrast can even be observed when scanning the cavity across a free spectral range of the cavity. These results open a path to cavity-enhanced measurements in applications where cavity length stabilization is impossible, i. e. in case the cavity is *non-length-stabilized*. Our continuous-wave approach will facilitate the usage of cavities for microscopy with the need for small spectral width, for example, the imaging of ultracold atoms.

**Fig. 1:**
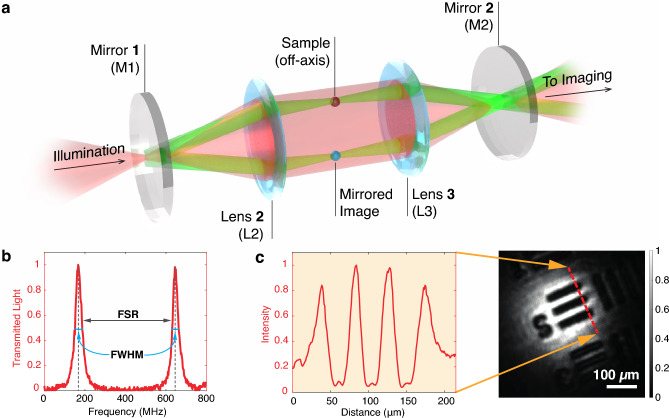
Cavity setup and characterization. (**a**) Ray tracing picture showing the self-imaging properties of the 4f cavity. The sample is illuminated with a collimated beam (red); the diffracted light (green) is imaged onto the sample after one full roundtrip. (**b**) Cavity length scan monitored by the output voltage (normalized) of a photodiode placed at the output of the cavity. The separation between the two resonance peaks yields a free spectral range (FSR) of $488\, \text {MHz}$ and a FWHM of $35.1\, \text {MHz}$ in that specific realisation (accordingly the finesse is 13.9; see Methods for Details). (**c**) We test the degeneracy of transversal modes by projecting the image of a US Air Force (USAF) Target onto the cavity image plane. The recorded light intensity in a conjugate plane after the cavity, and a cross-section thereof, demonstrate its image transmission capabilities.

**Fig. 2:**
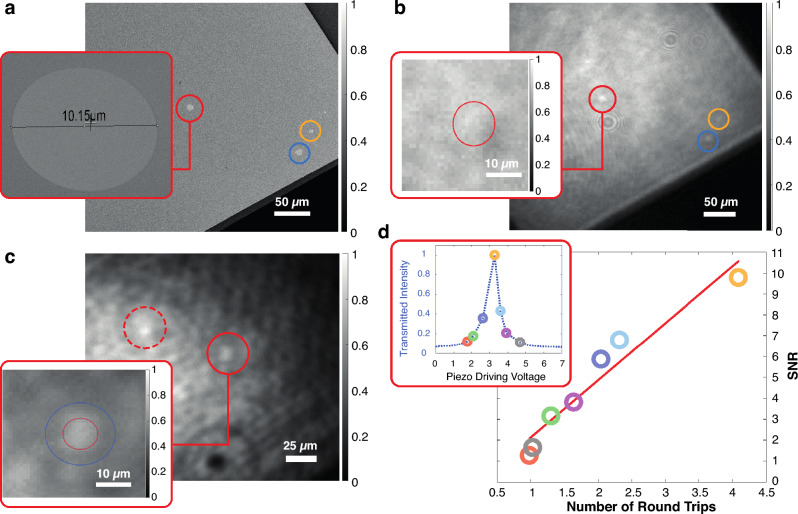
Cavity-enhanced microscopy of holes in a $\text {Si}_3\text {N}_4$ membrane. (**a**) Scanning electron microscopy image of holes cut into a $\text {Si}_3\text {N}_4$ membrane. The red and blue circles indicate holes with a diameter of $10\,\mu \text {m}$, and the yellow circle marks a hole with a diameter of $6\,\mu \text {m}$. The inset shows a close-up of the hole we use as our main target. (**b**) Defocused single-pass image of the membrane; the inset shows the region of the image with the target in focus; the target hole is not visible (see main text). (**c**) Cavity-enhanced image of the membrane; the two circles indicate the targeted hole (solid) and its mirror image (dashed line). The inset shows a close-up of the target; compared to the single pass case we find a contrast enhancement; the two circles show the regions for averaging signal (red) and background (blue without red) intensities, used to estimate contrast and SNR. (**d**) Signal-to-noise ratio in the cavity-enhanced case plotted against the number of effective round trips. The color-coded data points match those in the inset, where the transmitted intensity is shown as a function of the cavity length.

*Degenerate cavity setup* For cavity-enhanced microscopy, it is crucial that spatial information is accurately transmitted through the cavity system. Degeneracy can be achieved with multiple cavity arrangements^5,13^. For example, confocal or concentric cavities feature a certain degree of degeneracy of transversal modes, which leads to re-imaging of the fields after a certain number of roundtrips. For our experiments, we chose a cavity geometry that realizes an 8f transformation after a single roundtrip. It guarantees that the output field is equal to the input field, allowing for a straightforward interpretation of the resulting images.

We employ an effective 4f configuration using two highly reflective planar cavity mirrors and a pair of $f = 75\,\text {mm}$ biconvex lenses; a schematic of the setup is shown in Fig.1a (see Supplementary Material for details and^6^ for all details on the assembly, and testing of the cavity). The sample is positioned off-axis to prevent overlapping with the mirrored image; as a result, the image is projected onto the sample only after completing one full round trip. Control over the cavity length is achieved via a piezo ring mounted on the first mirror. For monitoring the cavity output, a photodiode is placed after a beamsplitter, and the remaining light is imaged on a charge-coupled device (CCD) camera. Various standard imaging modalities, such as bright-field, darkfield, or Zernicke phase imaging, can be implemented outside the cavity (see Supplementary Material for imaging details).

In Fig. 1b, we present a cavity scan where we show the output intensity measured using a photodiode as a function of the cavity length. The spacing between the two peaks represents the free spectral range (FSR), achieved by moving the mirror over half a wavelength $\lambda$. From the distance between the two peaks, we obtain an FSR of $488\, \text {MHz}$ and a FWHM of $35.1\, \text {MHz}$ (accordingly the finesse is 13.9), which aligns well with the theoretical predictions (see Supplementary Material for details).

To demonstrate image transmission capabilities, a US Air Force (USAF) target is positioned in the object plane of the focusing lens in front of the cavity. The resulting image is coupled into the self-imaging cavity and then captured by the CCD camera. As shown in Fig. 1c, the image clearly reveals the structure of the USAF target, confirming that many transverse modes are transmitted through the cavity.

*Theoretical results for cavity-enhancement* The theoretical performance of our approach is analyzed in a calculation that keeps track of phase shifts along each closed path within the cavity (see Supplementary Material). Our key performance indicators are the sample contrast and the signal-to-noise ratio (SNR) at fixed input power,

1 $$\begin{aligned} {\mathcal {C}} = \frac{|I_S - I_B|}{I_S + I_B}, \qquad \text {SNR} = \frac{|I_{S}-I_{B}|}{\sqrt{I_{S}+I_{B}}}, \end{aligned}$$

where $I_{S}$ is the output intensity measured at the sample position, and $I_{B}$ is the average output intensity measured next to the sample.

While in phase-contrast microscopy the contrast would be ${\mathcal {C}}_{PC} = 2|(\chi -\chi _0)|$^19^, we find for our cavity-enhanced approach:

2 $$\begin{aligned} {\mathcal {C}}_{\max }&\approx \frac{2\sqrt{R_2}}{T_1+T_2} |(\chi -\chi _0)\sin 4kf|, \end{aligned}$$

where $R_i$ and $T_i$ denote the reflectivity and transmission of the cavity mirrors $i\in {1,2}$, $\chi$ and $\chi _0$ are the weak phase shifts of the sample and a reference point, respectively, and $k=2\pi /\lambda$. Note that the prefactor is $>>1$ if high-reflectivity mirrors are used ($T_i<<1$).

Strikingly, when averaging over the resonance in the course of the measurement, the contrast is still enhanced compared to a single pass:

3 $$\begin{aligned} {\mathcal {C}}_\textrm{avg}&\approx \frac{\sqrt{R_2}}{T_1+T_2} |(\chi -\chi _0)\sin 4kf| = \frac{{\mathcal {C}}_{\max }}{2} , \end{aligned}$$

Note that in the shot-noise limited case the signal-to-noise ratio at constant damage SNR$_D$ will also be enhanced^12^.

This enhanced performance of an non-length-stabilized cavity relies on all modes within the cavity undergoing the same fluctuations. It can also be derived from a textbook-like toy model in which a Fabry-Pérot cavity is used for gas cell spectroscopy. If the input mirror vibrates across an FSR, cavity enhancement is still obtained, if the cavity output is interfered with a reference from an empty cavity that undergoes the same fluctuations in cavity length. We provide this calculation in the Method section, where we also show that the 2x loss in contrast for non-length-stabilized cavities is due to light being reflected from the cavity. Analyzing the reflected light, will regain the lost information.

**Fig. 3:**
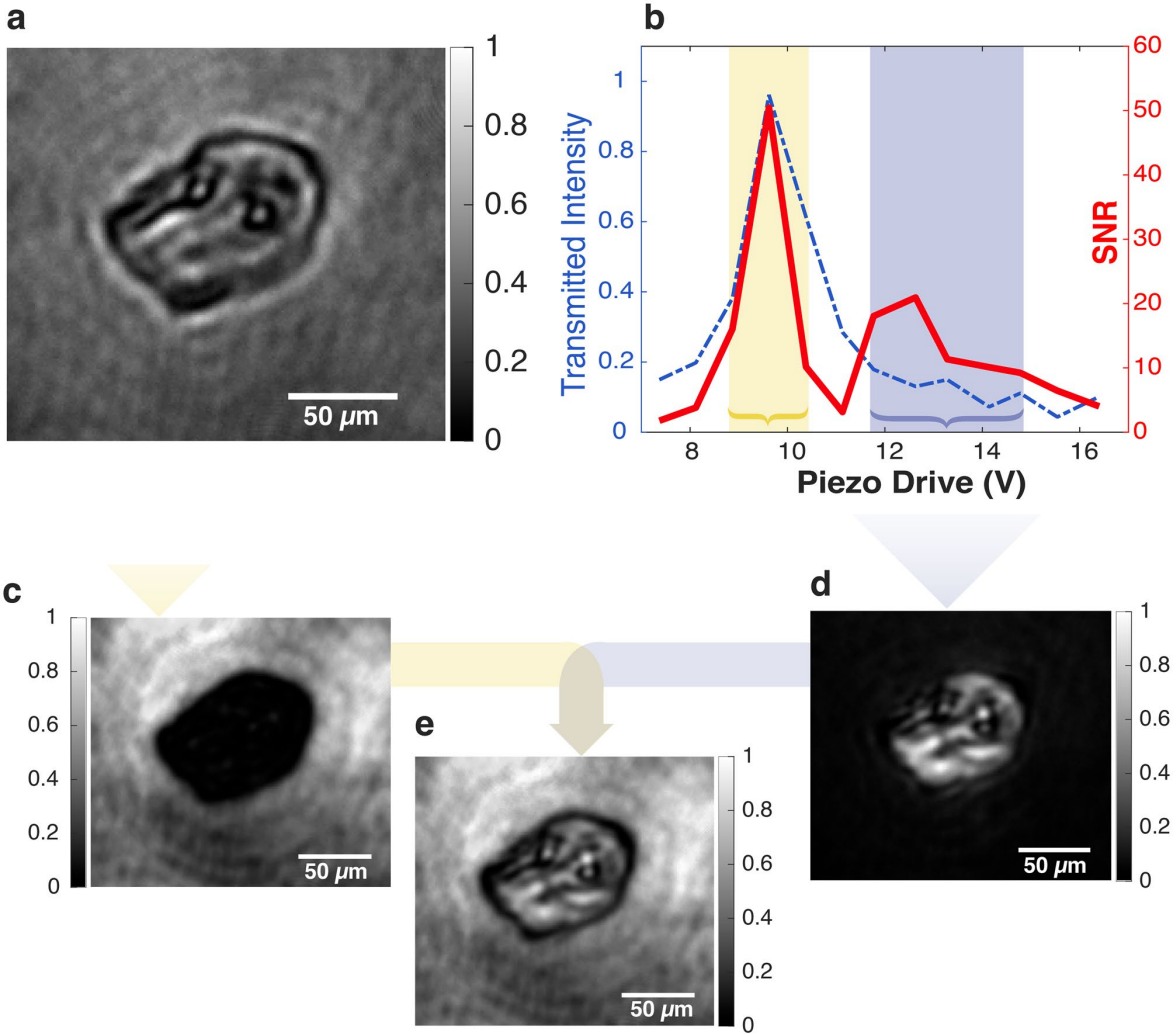
Cavity dark-field imaging. (**a**) Bright Field image of cheek cell in single pass. (**b**) Calculated SNR as a function of mirror displacement represented by the driving voltage of the piezo. The blue curve shows the cavity output detected by the photodiode. (**c**) Yellow: Integrated images over time (see Supplementary Material for all pictures), while the incidence light is resonant and the refracted light is out of resonance. (**d**) Dark Blue: Integrated images over time while incidence light is out of resonance and refracted light on resonance. This represents a novel approach to dark-field imaging. (**e**) Integrating over all the detunings, we obtain a picture corresponding to an non-length-stabilized cavity with increased contrast as compared to the single-pass image.

*Demonstration of cavity-enhanced microscopy* The enhancement of multi-pass techniques is closely linked to the effective number of round trips *N*^4,12,14^. For continuous-wave cavities, we can calculate the effective *N* by comparing the intensities inside and outside the cavity for a given cavity length,

4 $$\begin{aligned} N = \frac{\sqrt{I_\textrm{cav}}}{\sqrt{T_1 I_\textrm{in}}} = \frac{\sqrt{I_\textrm{out}}}{\sqrt{T_1 T_2 I_\textrm{in}}}. \end{aligned}$$

Measuring the total input and output intensities $I_\textrm{in}, I_\textrm{out}$ thus allows us to evaluate *N* for a given cavity setting in the experiment.

In order to demonstrate the contrast-enhancing capabilities of the setup, we cut holes with diameters of $6\ \& \ 10\,\mu \text {m}$ in a $10\,\text {nm}$ thin silicon nitride ($\text {Si}_3\text {N}_4$) membrane, which forms a $500\times 500\,\mu \text {m}$ window on a silicon frame; the membrane has a reflectance of approximately $1.5\,\%$. Fig.2a shows a scanning electron microscopy image of the structure. In Fig.2b we show a single-pass defocused image^20^, where the hole is visible as a bright spot. The inset shows a focused single-pass microscopy image in which the holes are not visible. This is consistent with the contrast levels expected for these techniques. While bright field-contrast would be based on reflection from the membrane and amounts to only $1.5\%$, defocus leads to phase contrast which can be as large as $\Delta I/I=2(\chi -\chi _0)=(n_\textrm{SiN}^2-1)kd=24\%$, where *d* is the thickness and $n_\textrm{SiN}$ denotes the index of refraction of $\text {Si}_3\text {N}_4$; see Supplementary Material.

We find a noticeable contrast enhancement in the cavity-enhanced image on resonance (Fig. 2c). To quantify the contrast, we averaged the intensity of the cavity-enhanced hole and compared it to the nearest averaged radial background. The calculated contrast was approximately $10\%$, considerably more than the single-pass bright-field contrast. We then evaluated the SNR for each captured image, plotting it against the effective number of roundtrips. Remarkably, we observed an enhanced SNR at the cavity resonance, as depicted in Fig.2d; the SNR overall follows the trend of the transmitted intensity (shown in inset of Fig. 2d). Further characterization of the image reveals a measured hole size of $\approx 11\,\mu {\text{m}}$ and a resolution of $3.05\mu {\text{m}}$, which aligns well with the expected values (see Supplementary Material), indicating that the cavity does not reduce the spatial resolution of our microscope.

*Microscopy of epithelial cells* Next, we image epithelial cells obtained from the inner lining of a human cheek, which are fixed onto an anti-reflection coated glass slide using methanol. Fig. 3a displays the morphology of these cells in a single-pass image, with the two black circles in the center likely representing the cell nuclei.

Performing cavity-enhanced microscopy, we first study the SNR as a function of cavity length, defining the whole cell as the signal region and the region around it as the background. We obtain a peak in SNR when the incident, unscattered background light is resonant (indicated by the yellow bar in Fig. 3). Notably, the transparent sample appears completely darkened (see Fig. 3c). Detuning the cavity, we observe a second “resonance” in the SNR when the cavity is off-resonant for the incident light. In Fig.3d, we show the resulting image, which clearly displays the cell content and its structures spatially resolved (see Supplementary Material for all images). The dispersive phase shift that the light undergoes while passing through the sample, along with the laser’s narrow bandwidth, is substantial enough for the refracted light to resonate at a different cavity length. This phenomenon introduces a new method for dark-field imaging; the peak shift can be used as a measure of the sample’s optical thickness.

*Microscopy with non-length-stabilized cavities* Finally, we show that signal amplification, and enhanced contrast, are also observed in an non-length-stabilized cavity. To demonstrate this effect experimentally, we integrate across the entire measurement, and obtain a combination of bright- and darkfield image, as shown in Fig.3e with enhanced contrast compared to single-pass. This effect is also achieved by integrating with the exposure time over one FSR scan. This makes our cavity-enhanced microscopy method applicable in scenarios without the possibility of stabilizing the cavity length actively.

*Conclusion and outlook.—* In this work, we demonstrate continuous-wave cavity-enhanced microscopy with a narrow laser bandwidth. We experimentally demonstrate increased contrast and signal-to-noise, and show both theoretically and experimentally that these advantages persist in non-length-stabilized cavities.

Furthermore, we present a new form of dark-field microscopy in which different regions of a sample, corresponding to different optical path lengths, light up at different cavity lengths. This corresponds to an entirely new contrast mechanism in optical microscopy. Contrary to traditional dark-field imaging, our scheme enables dark-field imaging with forward scattered light, which is ideal for quantitatively assessing samples characterized by slowly varying phase shifts.

A potential application of our method is cavity-enhanced imaging of ultracold atoms, which so far has only been done with single mode cavities^21^. Dispersive imaging, utilizing the phase shifts^22^, has been demonstrated to allow for multiple repeated images^23^. We propose using cavity-enhanced imaging to improve the signal-to-noise ratio at fixed feedback to the quantum system to lower the impact of e.g. heating^24^. The forward scattering leading to the phase signal is coherently amplified by the cavity, in contrast to incoherent scattering processes. This should enable high SNR dispersive imaging of ultracold atoms. At the same time, the imaging cavity provides new ways of applying local control fields within the cavity^25^.

## Methods

### Cavity parameters

The cavity length is defined by the 4*f* requirement to be $\sim 30\,\text {cm}$. Accounting for the optical path length differences introduced by the lenses results in an actual length of approximately $30.6\,\text {cm}$. This corresponds to an FSR of $493\,\text {MHz}$, which is in good accordance with the experimentally found value of $\approx 488\,\text {MHz}$. We use two highly reflective mirrors with $R_1 = 95\,\%$ and $R_2 = 86\,\%$, yielding a theoretical finesse value of 29.8.

We scan the cavity across the resonance by changing the cavity length with a piezo. We calibrate the applied voltage with a frequency reference. We do so by injecting two laser frequencies. The frequency difference is small compared to the FSR but larger than the FWHM of the cavity resonance and thus can be used as a ruler.

In the actual experimental environment, we observed fluctuating finesse values between 10 and 25. The presence of optical elements within the cavity and the sample carriers likely contributed to this reduction in overall finesse. We observed cavity length drifts of about $\pm 30\,\text {MHz}$ on the one second scale.

### Data analysis and error bars

The shown data points for the SNR are acquired from a single realization. Measuring at exactly the same cavity length would require length-stabilisation; we checked that the features shown in the main text are reproducible (see Supplementary Material figure for repeated scans).

### Enhancement with non-length-stabilized cavity

In the main text, we demonstrate cavity-enhanced microscopy of an optically thin lossless sample and show evidence that an enhancement of the phase contrast prevails even in the case of an non-length-stabilized cavity resonance. Here, we corroborate our finding with the help of an instructive toy model based on a Fabry-Pérot cavity - a setup known from textbooks and many applications. This simple example illustrates both the contrast enhancement and its robustness against cavity fluctuations without lengthy formulas.

**Fig. 4:**
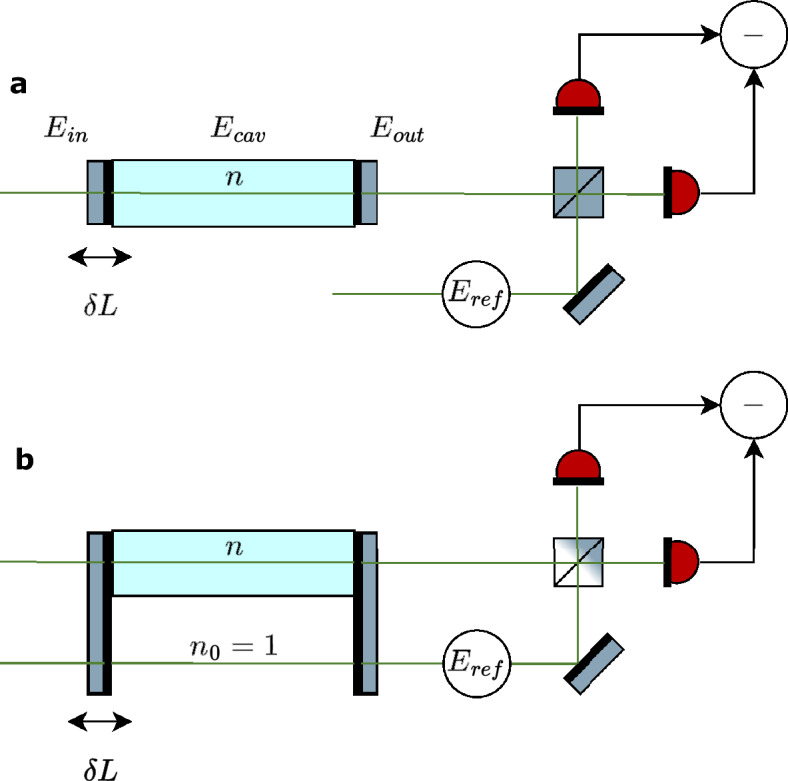
Set-up for toy model calculation. (**a**) The output of a Fabry-Pérot cavity setup is homodyned with a local oscillator. (**b**) Additionally, the local oscillator is transmitted through the same non-length-stabilized cavity picking up the phase shift of the detuned cavity. For both cases, we analyze the possible precision in estimating the phase shift imprinted by a thin lossless sample inside the cavity.

Consider a Fabry-Pérot cavity of length *L* with end mirrors of reflectivity $R = 1-T$ that contains a homogeneous, lossless, and optically thin medium. The task is to infer its weak refractive index *n* from a measurement of the small phase shift, $\chi = (n-1)k L \ll 1$, it imparts on a single-mode light field passing through the cavity. A balanced beam splitter then superimposes a phase-locked reference beam on this field, after which the intensities in the two output ports are detected; see Fig. 4. We will take their difference as the measurement signal.

Let us assume for now that the cavity length is stable. Given a monochromatic input field amplitude $E_\textrm{in}$ of wavenumber $k = 2\pi /\lambda$, the transmitted field reads as^26^

5 $$\begin{aligned} E_\textrm{out} = \frac{T E_\textrm{in} e^{ik(n-1)L}}{1 - R e^{2iknL} } \approx \sqrt{T}E_\textrm{cav} \left[ 1 + i\chi \frac{1+Re^{2ikL}}{1-Re^{2ikL}} \right] . \end{aligned}$$

Here and in the following, we consistently expand to lowest order in the phase shift $\chi$ we seek to estimate, and we denote the field amplitude inside the *empty* cavity as

6 $$\begin{aligned} E_\textrm{cav} = \frac{\sqrt{T} E_\textrm{in}}{1-R e^{2ikL}}. \end{aligned}$$

Notice that all field amplitudes are defined with respect to the position of the right cavity mirror on the optical axis, $z\equiv 0$.

We achieve perfect, resonant transmission if we adjust the cavity length or the light wavelength such that the optical path length 2*L* over one roundtrip is a multiple of the wavelength, $kL = \ell \pi$ with $\ell \in {\mathbb {N}}$. This leads to constructive interference enhancing both the intra-cavity field strength, $E_\textrm{cav}^\textrm{res} = E_\textrm{in}/\sqrt{T} > E_\textrm{in}$, and the response to the phase shift in (5),

7 $$\begin{aligned} E_\textrm{out}^\textrm{res} = \sqrt{T} E_\textrm{cav}^\textrm{res} \left[ 1 + i\chi \frac{1+R}{T}\right] . \end{aligned}$$

For phase-sensitive detection, we combine the output with a reference beam of, say, the same input field strength phase-shifted by $\pi /2$, $E_\textrm{ref} = iE_\textrm{in}$, as depicted in Fig. 4(a). The intensities detected after the beam splitter are given by $|E_\textrm{ref} \pm E_\textrm{out}|^2/4$, up to constant prefactors, and the measurement signal by the difference of these two intensities,

8 $$\begin{aligned} {\mathcal {S}}_\textrm{a}&\propto \textrm{Re} \left\{ E_\textrm{ref}^* E_\textrm{out} \right\} \nonumber \\&\approx T|E_\textrm{in}|^2 \textrm{Im} \left\{ \frac{1+i\chi \frac{1+Re^{2ikL}}{1-Re^{2ikL}}}{1-Re^{2ikL}} \right\} . \end{aligned}$$

The resonant case yields a gain in phase signal due to the enhancement of the intra-cavity intensity with 1/*T*,

9 $$\begin{aligned} {\mathcal {S}}^\textrm{res} \propto |E_\textrm{in}|^2 \frac{1+R}{T}\chi = T|E_\textrm{cav}^\textrm{res}|^2 \frac{1+R}{T}\chi . \end{aligned}$$

Not only the signal is enhanced on resonance, but also the signal-to-noise ratio (SNR): assuming shot-noise-limited detection, the standard deviation ${\mathcal {N}}$ of the measurement signal is proportional to the square-root of the sum of the two detected intensities. In the resonant case, ${\mathcal {N}}^\textrm{res} \propto |E_\textrm{in}|$, and hence the SNR improves with 1/*T*.

For sensitive specimens, it is important to characterize the SNR at a fixed intracavity intensity, i.e., a fixed amount of probe-induced specimen damage. To do so, we compare the cavity-enhanced case with a single pass scheme with an input power increased by $(1+R)/T$. We retrieve a signal-to-noise ratio at constant damage (SNR$_D$) that improves by $1/\sqrt{T}$^12^, indicating another fundamental advantage of cavity-enhanced measurements over single-pass measurements.

What happens if the cavity length *L* is unstable for the duration of the experiment? We can describe this by taking uniform averages with respect to *L* over one spectral range $\lambda /2$, neglecting the small variation of the phase shift $\chi$ over this range. Analytic expressions for the averaged intracavity intensity and the measurement signal are readily obtained with help of Mathematica,

10 $$\begin{aligned} \overline{|E_\textrm{cav}|^2}&= \frac{|E_\textrm{in}|^2}{1+R}, \end{aligned}$$

11 $$\begin{aligned} {\overline{{\mathcal {S}}}}_\textrm{a}&\propto T|E_\textrm{in}|^2 \chi = T \overline{|E_\textrm{cav}|^2} (1+R) \chi . \end{aligned}$$

The average intracavity intensity is no longer enhanced, but *lower* than the input intensity, reducing the signal proportionally. However, even if we increase the input power to match the intra-cavity intensity of the resonant signal (9), the sensitivity to the phase shift is still diminished, lacking the resonant gain by 1/*T*. In agreement with our intuition, we do not observe an enhanced signal, SNR, and SNR$_D$ with a cavity of unstable length.

However, the situation changes if we let the reference beam pass an empty region of the same cavity and thus be subject to the same unstable length as the probe field; see Fig. 4(b). This is described by the amplitude $E_\textrm{ref} = i\sqrt{T}E_\textrm{cav}$, which leads to the measurement signal

12 $$\begin{aligned} {\mathcal {S}}_\textrm{b} \propto T|E_\textrm{cav}|^2 \textrm{Re} \left\{ \chi \frac{1+Re^{2ikL}}{1-Re^{2ikL}}\right\} \end{aligned}$$

On resonance ($kL = \ell \pi$), the result is the same as before in (9). The length-averaged signal for an non-length-stabilized cavity now reads as

13 $$\begin{aligned} {\overline{{\mathcal {S}}}}_\textrm{b} \propto |E_\textrm{in}|^2 \frac{1+R^2}{(1+R)^2}\chi = T\overline{|E_\textrm{cav}|^2} \frac{1+R^2}{(1+R)T}\chi . \end{aligned}$$

Once again, the signal suffers from the reduced intra-cavity intensity, but at matching intensity, the gain in phase sensitivity persists. At high cavity finesse, $R\approx 1$, the sensitivity is reduced merely by a factor two compared to the resonant case (9). Regarding SNR, the detected intensities are diminished by *T* compared to the input, so that ${\mathcal {N}} \propto \sqrt{T}$ and hence SNR $\propto 1/\sqrt{T}$. Compared to a single pass, the intracavity intensity is now reduced by $1/(1+R) \approx 1/2$, while the detected intensity is reduced further by *T*. Hence, at matching damage, SNR$_D$ improves by $\sqrt{2/T}$.

In the Supplementary Material, we analyze an imaging scenario in which many transverse modes are enhanced in a self-imaging cavity. Since they all are transmitted through the same cavity, the assumption of having common noise on the cavity path length is justified for small path length changes on the order of $\lambda$ and in the paraxial limit. Spatially distributed phase shifts can be quantified using phase contrast microscopy, which can be analyzed as an interference effect between the scattered and unscattered light. The imaging setup thus plays a role similar to the homodyne detection in our toy model. Also there, we find enhanced signal contrast, which implies, by virtue of the same arguments as made here, that the SNR and SNR$_D$ are enhanced compared to single-pass imaging as well.

### Information in reflected field

We have seen that, in the limit of high cavity finesse, the length average leads to a loss in sensitivity by two in case (b) compared to the resonant result. The intuition is that this should come from the signal that is transmitted through the left cavity mirror. The total outgoing field from the left mirror to the left is

14 $$\begin{aligned} E_\textrm{out}^{\leftarrow } = \sqrt{R} E_\textrm{in} \left[ 1-\frac{Te^{2inkL}}{1-Re^{2inkL}} \right] = \frac{\sqrt{R} E_\textrm{in} (1-e^{2inkL})}{1-Re^{2inkL}}, \end{aligned}$$

and one can easily check the conservation of total energy flux, $|E_\textrm{in}|^2 = |E_\textrm{out}|^2+|E_\textrm{out}^{\leftarrow }|^2$. For weakly refractive media, we again expand to lowest order in $\chi$ and find,

15 $$\begin{aligned} E_\textrm{out}^{\leftarrow } \approx \sqrt{R}E_\textrm{in} \left[ \frac{1-e^{2ikL}}{1-Re^{2ikL}} - 2i\chi \frac{Te^{2ikL}}{(1-Re^{2ikL})^2} \right] . \end{aligned}$$

Phase-sensitive detection of this field with help of an empty reference cavity of the same varying length is achieved in the same manner as for the outgoing field on the right. Given the phase-shifted reference field $E_\textrm{ref}^{\leftarrow } = iE_\textrm{out}^{\leftarrow }|_{\chi =0}$ of the empty cavity, we have

16 $$\begin{aligned} {\mathcal {S}}_\textrm{b}^{\leftarrow }&\propto \textrm{Re} \left\{ E_\textrm{ref}^{\leftarrow *} E_\textrm{out}^{\leftarrow } \right\} \nonumber \\&= \frac{2T R (1+R) |E_\textrm{in}|^2}{|1-Re^{2ikL}|^4}(1-\cos 2kL)\chi . \end{aligned}$$

Averaging this over the length variation results in

17 $$\begin{aligned} \overline{{\mathcal {S}}_\textrm{b}^{\leftarrow }} \propto |E_\textrm{in}|^2 \frac{2R}{(1+R)^2}\chi . \end{aligned}$$

Adding up both average signals, we get $\overline{{\mathcal {S}}}_\textrm{b} + \overline{{\mathcal {S}}_\textrm{b}^{\leftarrow }} \propto |E_\textrm{in}|^2 \chi = \overline{|E_\textrm{cav}|^2} (1+R) \chi$. Hence, if we increase the input intensity to match the 1/*T*-enhanced intracavity intensity of the resonant case (9), then for $R\approx 1$ the total sensitivity to $\chi$ is the same. Indeed, the missing factor of two in the forward signal is recovered by also monitoring the output on the left.

## Supplementary Information

Supplementary Information 1.

## Data Availability

Source data and all other data that support the plots within this paper and other findings of this study are available from the corresponding author upon reasonable request.
