# Supplementary material for: Cavity-enhanced continuous-wave microscopy with potentially unstable cavity length
Source: Sci Rep. 2025 Jul 29;15:27676. doi: 10.1038/s41598-025-13589-w (PMC12307652; doi:10.1038/s41598-025-13589-w)
Supplement: Supplementary file 1 — Supplementary Information 1. [file 41598_2025_13589_MOESM1_ESM.pdf]

Cavity-enhanced continuous-wave microscopy with potentially unstable cavity length

Oliver Lueghamer,[1] Stefan Nimmrichter,[2] Clara Conrad-Billroth,[3,4] Thomas Juffmann,[3,4] and Maximilian Prüfer[1,*]

[1] *Vienna Center for Quantum Science and Technology, Atominstitut, TU Wien, Stadionallee 2, 1020 Vienna, Austria*
[2] *Naturwissenschaftlich-Technische Fakultät, Universität Siegen, Siegen 57068, Germany*
[3] *University of Vienna, Faculty of Physics, VCQ, A-1090 Vienna, Austria*
[4] *University of Vienna, Max Perutz Laboratories, Department of Structural and Computational Biology, A-1030 Vienna, Austria*

SUPPLEMENTARY MATERIAL

Experimental setup

In Fig. A1, we show the complete setup, consisting of three main parts. First, the 780 nm laser undergoes beam preparation, depicted at the top. Power regulation without altering the laser current is achieved using a half-wave plate ($\lambda/2$) and a polarizing beamsplitter (PBS). A double-pass acousto-optic modulator (AOM) adjusts the frequency, with ruler frequencies enabling free spectral range (FSR) measurement.

After passing through a single-mode fiber, the beam enters the core of the setup: the self-imaging cavity. A polarizing beam splitter (PBS) and a quarter-wave plate ($\lambda/4$) image reflections onto a CMOS camera for alignment. The cavity consists of two partially reflecting mirrors, M_1 and M_2, and two bi-convex lenses, L_2 and L_3, arranged in a $4f$ configuration. The beam is focused by L_1 onto M_1, collimated by L_2, and passes through a sample in the cavity center. Lens L_3 and a post-cavity lens L_4 create a telescopic system with a magnification of 6.6x. This output is imaged via another $4f$-setup, optionally including a phase plate and an externally triggered camera (see Fig. A2). Finally, a $70:30$ non-polarizing beamsplitter directs part of the signal to two photodiodes for monitoring.

In this work, we do not actively stabilize the cavity to a fixed length, but our setup allows for scanning across specific cavity resonances. The driving voltage, which reflects the displacement, and the output signal are continuously monitored. During the measurement, every 100 ms we record a microscopy image with the CCD camera.

Optical Components

We selected our mirrors based primarily on availability, cost-effectiveness, and their physical characteristics. They needed to be anti-reflective (AR) coated for $\lambda = 780$ nm and have sufficient reflectivity to support approximately $10-20$ round trips. The "Femtoline Laser Output Couplers" from EKSMA met these criteria perfectly. Specifically, we used the "EKSMA *045-1197*" with a reflectivity of $R_1 = 97 \pm 1\,\%$ as the in-coupling or front mirror (M_1) and the "EKSMA *045-1190*" with $R_2 = 90 \pm 2\,\%$ as the out-coupling or rear mirror (M_2). This setup was expected to yield a finesse of $\mathcal{F} = 45.1$ and support approximately $U \approx 15$ round trips.
However, these mirrors were optimized for a wavelength of 800 nm, so we had to verify the reflection coefficients for 780 nm. The correct values turned out to be $R_1 = 95\,\%$ and $R_2 = 86\,\%$, resulting in a finesse of $\mathcal{F} = 29.8$ and support for approximately $U \approx 10$ round trips.
To scan over the free spectral range of the cavity, we have to be able to tune its length in the range of $\frac{\lambda}{2}$. Therefore the in-coupling mirror is glued onto a piezoelectric ring ("Thorlabs *PA44M3KW*"), which is driven by an amplified signal from an arbitrary waveform generator (AWG). Our proof-of-principle setup relies exclusively on one-inch optics. Consequently, we opted for lenses with a focal length $f_{2,3} = 75$ mm to construct the 4f setup within the resonator, resulting in a resolution of 2.8 μm. These lenses are basic B-coated bi-convex lenses sourced from Thorlabs, specifically the "LB1901-B - N-BK7 Bi-Convex Lens".

* Corresponding author: maximilian.pruefer@tuwien.ac.at

Resolution in cavity-enhanced microscopy

To estimate the resolution, we analyze the holes while the cavity is resonant. We are approaching this by modeling the hole as a box function. Due to the Gaussian blur induced by the used optics, the walls of the box profile are blurred as well. Consequently, the resolution can be determined by convolving the box function with a Gaussian. The resulting blur is dependent on σ, providing a reliable estimate of the resolution achieved. Let $B(x)$ be a box function of width $2a$:

$$B(x) = \begin{cases} \frac{1}{2a} & \text{if } |x| \leq a \\ 0 & \text{otherwise} \end{cases}$$

and $G(x)$ the Gaussian function:

$$G(x) = \frac{1}{\sqrt{2\pi}\sigma} e^{-\frac{x^2}{2\sigma^2}}$$

with standard deviation σ. The convolution is then given by:

$$f(x) = \int_{-\infty}^{\infty} B(t)G(x-t)\,dt = \frac{1}{4a}\left[\text{erf}\left(\frac{x+a}{\sqrt{2}\sigma}\right) - \text{erf}\left(\frac{x-a}{\sqrt{2}\sigma}\right)\right]$$

and σ is found by fitting this model to the data of a hole intensity profile. The hole size was measured by looking at the intensity plot in Supplementary Material Fig. 3, which represents a cross section through the hole, yielding a standard deviation of $\sigma \approx 3\mu m$.

Cavity-enhanced bright-field imaging of an optically thin sample - full multimode case

Here we provide a linear-optics model for the bright-field signal of an optically thin, lossless sample in our 4f-cavity setup. We show that there is a gain in the contrast of the detected signal to local variations of the sample's phase shift if the imaging cavity is set to resonant transmission, as demonstrated in the main text for a 10 nm thin, punctured Si_3N_4 membrane. We also show that half of the contrast gain persists if the signal is averaged over a free spectral range of the imaging cavity, representing a scenario in which the cavity mirror positions are unstable over the course of the measurement.

The 4f-cavity setup and model assumptions

The cavity setup is depicted in Supplementary Material Fig. 2. It consists of two mirrors M1 and M2 with reflectivities R_1 and R_2 and two thin lenses of focal length f in between, in a $4f$-arrangement. The position of mirror M1 can deviate by a few wavelengths $\lambda \ll f$ from its ideal position in the focal plane of the first lens. The sample, positioned in the central focus plane of both lenses, shall be described by a two-dimensional structure with thickness d_s and real-valued refractive index $n_s(\boldsymbol{r}_\perp)$. We will be concerned with spatial variations above the diffraction limit and assume a constant sample thickness; holes in the sample are represented by pixels with $n_s(\boldsymbol{r}_\perp) = 1$ Here and throughout, $\boldsymbol{r}_\perp = (x, y)$ denotes the transverse coordinates on the any plane perpendicular to the optical axis, and z denotes the optical axis coordinate. We make the following assumptions:

- The light that probes the sample is a continuous-wave field (or sufficiently long pulse) of wavelength λ in the paraxial regime, as described by a complex electric field amplitude $E(\boldsymbol{r}_\perp)e^{ikz}$ with wave number $k = 2\pi/\lambda$. We will omit the complex exponent and state all field amplitudes with respect to a reference plane at $z \equiv 0$ (say, the left focal plane of the first intracavity lens).

- For the reflection coefficients of the two mirrors M1 and M2, we choose the convention that the electric field interferes destructively with its reflection on the respective surfaces facing *inside* the cavity, $r_{1,2} = -\sqrt{R_{1,2}}$. The reflection coefficients on the outside surfaces are thus of opposite, positive sign, and the transmission coefficients are $t_{1,2} = \sqrt{1 - R_{1,2}}$.

- The lenses are assumed to be ideally thin, and we neglect any resolution limit given by their aperture or other imperfections. Given the electric field profile $E(\boldsymbol{r}_\perp)$ illuminating the lens from one focal plane, the image on

the opposite focal plane is then given by a Fourier transformation,

$$E_{2f}(\boldsymbol{r}_\perp) = \frac{-ik}{2\pi f} e^{2ikf} \widetilde{E}\left(\frac{k}{f}\boldsymbol{r}_\perp\right), \qquad \text{with} \quad \widetilde{E}(\boldsymbol{q}) = \int \mathrm{d}^2 r_\perp E(\boldsymbol{r}_\perp) e^{-i\boldsymbol{q}\cdot\boldsymbol{r}_\perp}. \tag{1}$$

A strongly focused spot transforms into a wide collimated beam and vice versa. A sequence of two $2f$-transformations (as e.g. experienced by the field reflected off a cavity mirror) results in an inversion of the field amplitude,

$$E_{4f}(\boldsymbol{r}_\perp) = -\left(\frac{k}{2\pi f}\right)^2 e^{4ikf} \widetilde{\widetilde{E}}\left(\frac{k}{f}\boldsymbol{r}_\perp\right) = -e^{4ikf} E(-\boldsymbol{r}_\perp). \tag{2}$$

- Given a paraxial beam and a free-standing sample structure sufficiently coarse compared to the light wavelength so that the imaging resolution is not limited by diffraction, we can describe the sample response by the position-dependent reflection and transmission coefficients of a dielectric slab with varying refractive index $n_s(\boldsymbol{r}_\perp)$,

$$t(\boldsymbol{r}_\perp) = \frac{4n_s(\boldsymbol{r}_\perp) e^{i[n_s(\boldsymbol{r}_\perp)-1]kd_s(\boldsymbol{r}_\perp)}}{[n_s(\boldsymbol{r}_\perp)+1]^2 - [n_s(\boldsymbol{r}_\perp)-1]^2 e^{2in_s(\boldsymbol{r}_\perp)kd_s(\boldsymbol{r}_\perp)}}, \tag{3}$$

$$r(\boldsymbol{r}_\perp) = \frac{[n_s^2(\boldsymbol{r}_\perp)-1] e^{-ikd_s(\boldsymbol{r}_\perp)} \left[e^{2in_s(\boldsymbol{r}_\perp)kd_s(\boldsymbol{r}_\perp)} - 1\right]}{[n_s(\boldsymbol{r}_\perp)+1]^2 - [n_s(\boldsymbol{r}_\perp)-1]^2 e^{2in_s(\boldsymbol{r}_\perp)kd_s(\boldsymbol{r}_\perp)}}. \tag{4}$$

  Notice that $r(\boldsymbol{r}_\perp)$ is the same for reflection off both sides.

- The input field $E_{\mathrm{in}}(\boldsymbol{r}_\perp)$, defined as the amplitude illuminating the mirror M1 from the left, shall illuminate the relevant parts of the sample more or less homogeneously. In particular, we assume that the illumination is spatially symmetric, $E_{\mathrm{in}}(\boldsymbol{r}_\perp) = E_{\mathrm{in}}(-\boldsymbol{r}_\perp)$.

- We allow the left mirror position to deviate from the focal plane by $\delta z_1 \sim \lambda$, but we neglect the influence of the shift on the imaging of the field (since $\delta z_1 \ll f$). That is, the shift will change the (empty) cavity length to $kL = 4kf - \phi_1$ with $\phi_1 = k\delta z_1$ influencing the cavity resonance; however, the shift is still small enough to neglect any defocusing caused by it. Notice that the shift also implies that the sample is no longer in the cavity center, but rather displaced by $-\delta z_1/2$.

Derivation of the output field and detection signal

Let $E_{\mathrm{in}}(\boldsymbol{r}_\perp)$ be the input field amplitude outside the left mirror M1, on the focal plane of the left intracavity lens. Including the phase shift $\phi_1 = k\delta z_1$ due to the mirror displacement by δz_1, and given the yet to be calculated backward-running wave from the sample that is reflected off M1 from the right, $E_{1\leftarrow}(\boldsymbol{r}_\perp)$, the forward-running wave field on the right of M1 is then the sum of the transmitted and the reflected components,

$$E_{1\rightarrow}(\boldsymbol{r}_\perp) = t_1 E_{\mathrm{in}}(\boldsymbol{r}_\perp) e^{i\phi_1} + r_1 E_{1\leftarrow}(\boldsymbol{r}_\perp). \tag{5}$$

The two running-wave fields on the right side of M1 are related to the running-wave fields on the left side of the sample plane via the $2f$-transform (1) and a phase shift by $-\phi_1$ due to the slightly shorter or longer distance $2f - \delta z_1$,

$$E_{\mathrm{L}\rightarrow}(\boldsymbol{r}_\perp) = -\frac{ik}{2\pi f} e^{2ikf - i\phi_1} \widetilde{E}_{1\rightarrow}\left(\frac{k}{f}\boldsymbol{r}_\perp\right), \qquad E_{1\leftarrow}(\boldsymbol{r}_\perp) = -\frac{ik}{2\pi f} e^{2ikf - i\phi_1} \widetilde{E}_{\mathrm{L}\leftarrow}\left(\frac{k}{f}\boldsymbol{r}_\perp\right). \tag{6}$$

Putting (5) and (6) together and invoking (2), we have

$$E_{\mathrm{L}\rightarrow}(\boldsymbol{r}_\perp) = E_0(\boldsymbol{r}_\perp) - r_1 e^{4ikf - 2i\phi_1} E_{\mathrm{L}\leftarrow}(-\boldsymbol{r}_\perp), \qquad \text{with} \quad E_0(\boldsymbol{r}_\perp) := -\frac{ikt_1}{2\pi f} e^{2ikf} \widetilde{E}_{\mathrm{in}}\left(\frac{k}{f}\boldsymbol{r}_\perp\right). \tag{7}$$

To the right of the sample plane, we can again relate the running-wave components to the ones left of M2 through a $2f$-transform. The difference is that there is no input field impinging on M2 from the right, so that $E_{2\leftarrow}(\boldsymbol{r}_\perp) = r_2 E_{2\rightarrow}(\boldsymbol{r}_\perp)$ and $E_{2\rightarrow}(\boldsymbol{r}_\perp) = (-ik/2\pi f) e^{2ikf} \widetilde{E}_{\mathrm{R}\rightarrow}(k\boldsymbol{r}_\perp/f)$. From this follows the relation between the waves right of the sample plane and the expression for the output field that we detect,

$$E_{\mathrm{R}\leftarrow}(\boldsymbol{r}_\perp) = -r_2 e^{4ikf} E_{\mathrm{R}\rightarrow}(-\boldsymbol{r}_\perp), \qquad E_{\mathrm{out}}(\boldsymbol{r}_\perp) = -\frac{ikt_2}{2\pi f} e^{2ikf} \widetilde{E}_{\mathrm{R}\rightarrow}\left(\frac{k}{f}\boldsymbol{r}_\perp\right). \tag{8}$$

In matrix notation, we can combine (7) and (8) into

$$\begin{bmatrix} E_{\mathrm{L}\rightarrow}(\boldsymbol{r}_\perp) \\ E_{\mathrm{R}\leftarrow}(\boldsymbol{r}_\perp) \end{bmatrix} = \begin{bmatrix} E_0(\boldsymbol{r}_\perp) \\ 0 \end{bmatrix} - e^{4ikf} \begin{bmatrix} 0 & r_1 e^{-2i\phi_1} \\ r_2 & 0 \end{bmatrix} \begin{bmatrix} E_{\mathrm{R}\rightarrow}(-\boldsymbol{r}_\perp) \\ E_{\mathrm{L}\leftarrow}(-\boldsymbol{r}_\perp) \end{bmatrix}. \tag{9}$$

The sample can be described by a transformation matrix mapping the incoming to the outgoing field components according to the coefficients (3) and (4),

$$\begin{bmatrix} E_{\mathrm{R}\rightarrow}(\boldsymbol{r}_\perp) \\ E_{\mathrm{L}\leftarrow}(\boldsymbol{r}_\perp) \end{bmatrix} = \mathsf{S}(\boldsymbol{r}_\perp) \begin{bmatrix} E_{\mathrm{L}\rightarrow}(\boldsymbol{r}_\perp) \\ E_{\mathrm{R}\leftarrow}(\boldsymbol{r}_\perp) \end{bmatrix}, \qquad \text{with} \quad \mathsf{S}(\boldsymbol{r}_\perp) = \begin{bmatrix} t(\boldsymbol{r}_\perp) & r(\boldsymbol{r}_\perp) \\ r(\boldsymbol{r}_\perp) & t(\boldsymbol{r}_\perp) \end{bmatrix}. \tag{10}$$

Notice that the matrix is unitary since $|t|^2 + |r|^2 = 1$ and $r^* t + r t^* = 0$, as one can easily check. For clarity and brevity of notation, we shall now drop the argument $\boldsymbol{r}_\perp$ and denote the coefficients and the matrix at this position by t, r, and S, whereas we denote by $\bar{t}, \bar{r}$ and $\bar{\mathsf{S}}$ the respective terms at the opposite position $-\boldsymbol{r}_\perp$. Moreover, we subsume $\tilde{r}_1 \equiv r_1 e^{-2i\phi_1}$. Plugging the sample transformation for $-\boldsymbol{r}_\perp$ into (9) and iterating the equation with our condition of symmetric illumination, $E_0(-\boldsymbol{r}_\perp) = E_0(\boldsymbol{r}_\perp)$, we obtain

$$\begin{aligned}
\begin{bmatrix} E_{\mathrm{L}\rightarrow}(\boldsymbol{r}_\perp) \\ E_{\mathrm{R}\leftarrow}(\boldsymbol{r}_\perp) \end{bmatrix} &= \begin{bmatrix} E_0(\boldsymbol{r}_\perp) \\ 0 \end{bmatrix} - e^{4ikf} \begin{bmatrix} 0 & \tilde{r}_1 \\ r_2 & 0 \end{bmatrix} \bar{\mathsf{S}} \begin{bmatrix} E_{\mathrm{L}\rightarrow}(-\boldsymbol{r}_\perp) \\ E_{\mathrm{R}\leftarrow}(-\boldsymbol{r}_\perp) \end{bmatrix} \\
&= \left(\mathbb{1} - e^{4ikf} \begin{bmatrix} 0 & \tilde{r}_1 \\ r_2 & 0 \end{bmatrix} \bar{\mathsf{S}} \right) \begin{bmatrix} E_0(\boldsymbol{r}_\perp) \\ 0 \end{bmatrix} + e^{8ikf} \begin{bmatrix} 0 & \tilde{r}_1 \\ r_2 & 0 \end{bmatrix} \bar{\mathsf{S}} \begin{bmatrix} 0 & \tilde{r}_1 \\ r_2 & 0 \end{bmatrix} \mathsf{S} \begin{bmatrix} E_{\mathrm{L}\rightarrow}(\boldsymbol{r}_\perp) \\ E_{\mathrm{R}\leftarrow}(\boldsymbol{r}_\perp) \end{bmatrix} \\
&= E_0(\boldsymbol{r}_\perp) \begin{bmatrix} 1 - e^{4ikf} \tilde{r}_1 \bar{r} \\ -e^{4ikf} r_2 \bar{t} \end{bmatrix} + e^{8ikf} \begin{bmatrix} \tilde{r}_1(\tilde{r}_1 \bar{r} r + r_2 \bar{t} t) & \tilde{r}_1(\tilde{r}_1 \bar{r} t + r_2 \bar{t} r) \\ r_2(\tilde{r}_1 \bar{t} r + r_2 \bar{r} t) & r_2(\tilde{r}_1 \bar{t} t + r_2 \bar{r} r) \end{bmatrix} \begin{bmatrix} E_{\mathrm{L}\rightarrow}(\boldsymbol{r}_\perp) \\ E_{\mathrm{R}\leftarrow}(\boldsymbol{r}_\perp) \end{bmatrix}.
\end{aligned} \tag{11}$$

The resulting linear equations can be solved straightforwardly,

$$\begin{aligned}
E_{\mathrm{L}\rightarrow} &= E_0 \frac{1 - e^{4ikf}[\tilde{r}_1 \bar{r} + e^{4ikf} r_2 (r_2 \bar{r} r + \tilde{r}_1 \bar{t} t) + e^{8ikf} \tilde{r}_1 r_2^2 r (\bar{t}^2 - \bar{r}^2)]}{1 - e^{8ikf}[(\tilde{r}_1^2 + r_2^2)\bar{r} r + 2\tilde{r}_1 r_2 \bar{t} t] + e^{16ikf} \tilde{r}_1^2 r_2^2 (\bar{t}^2 - \bar{r}^2)(t^2 - r^2)}, \\
E_{\mathrm{R}\leftarrow} &= E_0 \frac{e^{4ikf} r_2 [-\bar{t} + e^{4ikf}(\tilde{r}_1 \bar{t} r + r_2 \bar{r} t) + e^{8ikf} \tilde{r}_1 r_2 t (\bar{t}^2 - \bar{r}^2)]}{1 - e^{8ikf}[(\tilde{r}_1^2 + r_2^2)\bar{r} r + 2\tilde{r}_1 r_2 \bar{t} t] + e^{16ikf} \tilde{r}_1^2 r_2^2 (\bar{t}^2 - \bar{r}^2)(t^2 - r^2)}.
\end{aligned} \tag{12}$$

where we have dropped the argument $\boldsymbol{r}_\perp$ of the field amplitudes, too. Another application of S leaves us with

$$\begin{aligned}
E_{\mathrm{R}\rightarrow} &= E_0 \frac{t - e^{4ikf}[r_2 \bar{t} r + \tilde{r}_1 \bar{r} t + e^{4ikf} \tilde{r}_1 r_2 \bar{t} (t^2 - r^2)]}{1 - e^{8ikf}[(\tilde{r}_1^2 + r_2^2)\bar{r} r + 2\tilde{r}_1 r_2 \bar{t} t] + e^{16ikf} \tilde{r}_1^2 r_2^2 (\bar{t}^2 - \bar{r}^2)(t^2 - r^2)}, \\
E_{\mathrm{L}\leftarrow} &= E_0 \frac{r - e^{4ikf}[r_2 \bar{t} t + \tilde{r}_1 \bar{r} r - e^{4ikf} r_2^2 \bar{r} (t^2 - r^2) - e^{8ikf} r_2^2 \tilde{r}_1 (\bar{t}^2 - \bar{r}^2)(t^2 - r^2)]}{1 - e^{8ikf}[(\tilde{r}_1^2 + r_2^2)\bar{r} r + 2\tilde{r}_1 r_2 \bar{t} t] + e^{16ikf} \tilde{r}_1^2 r_2^2 (\bar{t}^2 - \bar{r}^2)(t^2 - r^2)}.
\end{aligned} \tag{13}$$

The output field in (8) that leaves the cavity on the right undergoes another transformation before it is detected: it passes a magnification lens L4 of focal length f_4 and, optionally, another $4f$-imaging system for further manipulation. For completeness, let us perform the $2f$-transformation corresponding to L4 and an unmanipulated 4f-transformation corresponding to the imaging system with f_5, which yields the magnified bright-field image of the sample,

$$\begin{aligned}
E_{\mathrm{bf}}(\boldsymbol{r}_\perp) &= -e^{4ikf_5} E_4(-\boldsymbol{r}_\perp) = \frac{ik}{2\pi f_4} e^{4ikf_5 + 2ikf_4} \int \mathrm{d}^2 \boldsymbol{r}'_\perp E_{\mathrm{out}}(\boldsymbol{r}'_\perp) e^{ik\boldsymbol{r}_\perp \cdot \boldsymbol{r}'_\perp / f_4} \\
&= \frac{k^2 t_2}{4\pi^2 f f_4} e^{4ikf_5 + 2ikf_4 + 2ikf} \int \mathrm{d}^2 \boldsymbol{r}'_\perp \mathrm{d}^2 \boldsymbol{r}''_\perp E_{\mathrm{R}\rightarrow}(\boldsymbol{r}''_\perp) e^{ik\boldsymbol{r}_\perp \cdot \boldsymbol{r}'_\perp / f_4 - ik\boldsymbol{r}'_\perp \cdot \boldsymbol{r}''_\perp / f} \\
&= \frac{t_2 f}{f_4} e^{4ikf_5 + 2ikf_4 + 2ikf} E_{\mathrm{R}\rightarrow}\left(\frac{f}{f_4} \boldsymbol{r}_\perp \right).
\end{aligned} \tag{14}$$

Since the magnification and the prefactors do not change the relevant phase signal of the sample, we will them implicitly and simply write $E_{\mathrm{bf}} = C E_{\mathrm{R}\rightarrow}$.

Linear response and signal of a weak sample

We get a clear picture about the cavity enhancement if we consider a weak and thin sample, i.e., we expand the sample coefficients of a thin slab to first order in $n_s k d_s$,

$$r(\boldsymbol{r}_\perp) \approx i\chi(\boldsymbol{r}_\perp), \quad t(\boldsymbol{r}_\perp) \approx 1 + i\chi(\boldsymbol{r}_\perp), \qquad \text{with} \quad \chi(\boldsymbol{r}_\perp) = \frac{n_s^2(\boldsymbol{r}_\perp) - 1}{2} k d_s \ll 1. \tag{15}$$

For our purposes, only the first, leading order in χ and $\bar{\chi} = \chi(-\boldsymbol{r}_\perp)$ is relevant, which allows us to neglect all second-order reflection terms $(r^2, \bar{r}^2, r\bar{r})$ in (12) and (13),

$$\begin{bmatrix} E_{\mathrm{L}\rightarrow} \\ E_{\mathrm{R}\leftarrow} \end{bmatrix} \approx \frac{E_0}{(1 - e^{8ikf}\tilde{r}_1 r_2 \bar{t}t)^2} \begin{bmatrix} 1 - e^{8ikf}\tilde{r}_1 r_2 \bar{t}t - e^{4ikf}\tilde{r}_1(\bar{r} + e^{8ikf} r_2^2 r \bar{t}^2) \\ e^{4ikf} r_2[-\bar{t}(1 - e^{8ikf}\tilde{r}_1 r_2 \bar{t}t) + e^{4ikf}(\tilde{r}_1 \bar{t} r + r_2 \bar{r} t)] \end{bmatrix}, \tag{16}$$

$$\begin{bmatrix} E_{\mathrm{R}\rightarrow} \\ E_{\mathrm{L}\leftarrow} \end{bmatrix} \approx \frac{E_0}{(1 - e^{8ikf}\tilde{r}_1 r_2 \bar{t}t)^2} \begin{bmatrix} t(1 - e^{8ikf}\tilde{r}_1 r_2 \bar{t}t) - e^{4ikf}(r_2 \bar{t} r + \tilde{r}_1 \bar{r} t) \\ r - e^{4ikf} r_2 \bar{t}t(1 - e^{8ikf}\tilde{r}_1 r_2 \bar{t}t) + e^{8ikf} r_2^2 \bar{r} t^2 \end{bmatrix}. \tag{17}$$

Recall that these are the fields evaluated at $+\boldsymbol{r}_\perp$ on the sample plane, but we obtain the fields at $-\boldsymbol{r}_\perp$ from the same formula if we exchange $r, t \leftrightarrow \bar{r}, \bar{t}$.

In order to see how the cavity can enhance the bright-field contrast of the sample response, we expand the relevant output field (14) to first joint order in χ and $\bar{\chi}$,

$$\begin{aligned} E_{\mathrm{bf}} = CE_{\mathrm{R}\rightarrow} &\approx \frac{E_0}{1 - e^{8ikf}\tilde{r}_1 r_2} \left[1 + \frac{1 - e^{4ikf} r_2}{1 - e^{8ikf}\tilde{r}_1 r_2} i(\chi - e^{4ikf}\tilde{r}_1 \bar{\chi}) \right] \\ &= \frac{E_0}{1 - e^{8ikf - 2i\phi_1}\sqrt{R_1 R_2}} \left[1 + \frac{1 + e^{4ikf}\sqrt{R_2}}{1 - e^{8ikf - 2i\phi_1}\sqrt{R_1 R_2}} i(\chi + e^{4ikf - 2i\phi_1}\sqrt{R_1}\bar{\chi}) \right], \end{aligned} \tag{18}$$

where we have used our convention for $r_{1,2} = -\sqrt{R_{1,2}}$. The outer prefactor describes the enhanced intra-cavity field strength, and it appears once more as a prefactor to the sample response. The prefactor achieves its maximum $(1 - \sqrt{R_1 R_2})^{-1}$ when the mirrors are perfectly positioned such that the cavity length $kL = 4kf - \phi_1 = \ell\pi$ with $\ell \in \mathbb{N}$. For the parameters in the manuscript, $R_1 = 0.95$ and $R_2 = 0.86$, this results in an amplification of about 10. The bright-field amplitude and intensity are then, to leading order,

$$E_{\mathrm{bf}}^{\max} = \frac{CE_0}{1 - \sqrt{R_1 R_2}} \left[1 + i\frac{\chi + \sqrt{R_1 R_2}\bar{\chi} + (-)^\ell (e^{i\phi_1}\sqrt{R_2}\chi + e^{-i\phi_1}\sqrt{R_1}\bar{\chi})}{1 - \sqrt{R_1 R_2}} \right], \tag{19}$$

$$|E_{\mathrm{bf}}^{\max}|^2 = \frac{|CE_0|^2}{(1 - \sqrt{R_1 R_2})^2} \left[1 + 2(-)^\ell \frac{\sqrt{R_1}\bar{\chi} - \sqrt{R_2}\chi}{1 - \sqrt{R_1 R_2}} \sin\phi_1 \right] = \frac{|CE_0|^2}{(1 - \sqrt{R_1 R_2})^2} \left[1 + 2\frac{\sqrt{R_1}\bar{\chi} - \sqrt{R_2}\chi}{1 - \sqrt{R_1 R_2}} \sin 4kf \right]. \tag{20}$$

This shows us that, in order to observe the optimal cavity-enhanced contrast for a purely phase-shifting sample, the mirror position δz_1 and the focal length f must be tailored precisely such that both $kL = 4kf - \phi_1 = \ell\pi$ and $\sin\phi_1 = (-)^\ell \sin 4kf = \pm 1$. This implies that both $4kf$ and ϕ_1 must be *odd* multiples of $\pi/2$, such that $\tilde{r}_1 = +\sqrt{R_1}$ and the sample displacement from the cavity center is an odd multiple of $\lambda/8$. In practice, it is unlikely that one achieves this precise balance since one typically does not even know the focal length of the lenses on the wavelength level and one thus cannot position the sample precisely enough in between the lenses either. On the other hand, it is also very unlikely that the configuration happens to be such that $4kf$ is a multiple of π and the first order response vanishes. In each of our measurements, we can therefore assume that $4kf$ takes some fixed unknown value in between; adjusting the mirror position for maximum overall bright-field intensity then amounts to setting $\phi_1 = 4kf - \ell\pi$.

In a non-length-stabilized cavity, the phase shift ϕ_1 can be random as the cavity mirrors are drifting or fluctuating in position. We can take this into account by averaging the bright-field intensity uniformly over ϕ_1 or, equivalently, over kL at fixed $4kf$. For given kL, ϕ_1, the intensity associated to (18) is

$$\begin{aligned} |E_{\mathrm{bf}}|^2 =& \frac{|CE_0|^2}{1 + R_1 R_2 - 2\sqrt{R_1 R_2}\cos 2kL} \left[1 - 2\mathrm{Im}\frac{(1 + e^{ikL + i\phi_1}\sqrt{R_2})(\chi + e^{ikL - i\phi_1}\sqrt{R_1}\bar{\chi})(1 - e^{-2ikL}\sqrt{R_1 R_2})}{1 + R_1 R_2 - 2\sqrt{R_1 R_2}\cos 2kL} \right] \\ =& \frac{|CE_0|^2}{1 + R_1 R_2 - 2\sqrt{R_1 R_2}\cos 2kL} \\ &\times \left[1 - 2\frac{(\chi + \bar{\chi})\sqrt{R_1 R_2}\sin 2kL + \sqrt{R_1}\sin(2kL - 4kf)(\bar{\chi} + R_2\chi) + \sqrt{R_2}(\chi + R_1\bar{\chi})\sin 4kf}{1 + R_1 R_2 - 2\sqrt{R_1 R_2}\cos 2kL} \right] \end{aligned} \tag{21}$$

Averaging this π-periodic function of kL uniformly over its period results in

$$\begin{aligned} \overline{|E_{\mathrm{bf}}|^2} &= \frac{|CE_0|^2}{1 - R_1 R_2} \left[1 - 2\frac{(1 + R_1 R_2)\sqrt{R_2}(\chi + R_1\bar{\chi})\sin 4kf - 2R_1\sqrt{R_2}(\bar{\chi} + R_2\chi)\sin 4kf}{(1 - R_1 R_2)^2} \right] \\ &= \frac{|CE_0|^2}{1 - R_1 R_2} \left[1 - 2\sqrt{R_2}\frac{\chi - R_1\bar{\chi}}{1 - R_1 R_2}\sin 4kf \right] \end{aligned} \tag{22}$$

In our experiment, we can quantify the image contrast by comparing the output intensities of a relevant sample pixel for which $\chi \neq \bar{\chi} \equiv \chi_0$ and a reference pixel with $\chi = \bar{\chi} = \chi_0$. In our case, χ_0 is the phase response of the Si_3N_4

membrane, whereas χ can be zero if we pick one of the holes in the membrane as our sample area. Taking as the contrast the magnitude of the difference in pixel intensities divided by the sum, we get at fixed $4kf$,

$$\mathcal{C}_{\rm max} \approx \frac{\sqrt{R_2}}{1-\sqrt{R_1 R_2}}|(\chi-\chi_0)\sin 4kf| \approx \frac{2\sqrt{R_2}}{T_1+T_2}|(\chi-\chi_0)\sin 4kf|, \tag{23}$$

$$\mathcal{C}_{\rm avg} \approx \frac{\sqrt{R_2}}{1-R_1 R_2}|(\chi-\chi_0)\sin 4kf| \approx \frac{\sqrt{R_2}}{T_1+T_2}|(\chi-\chi_0)\sin 4kf|, \tag{24}$$

to lowest order for the ideal case (20) and for the averaged case (22), respectively. On the right, we have also expanded to lowest order in the mirror transmission, $T_{1,2} = 1 - R_{1,2} \ll 1$, which reveals a mere factor-two difference between the ideal and the averaged contrast.

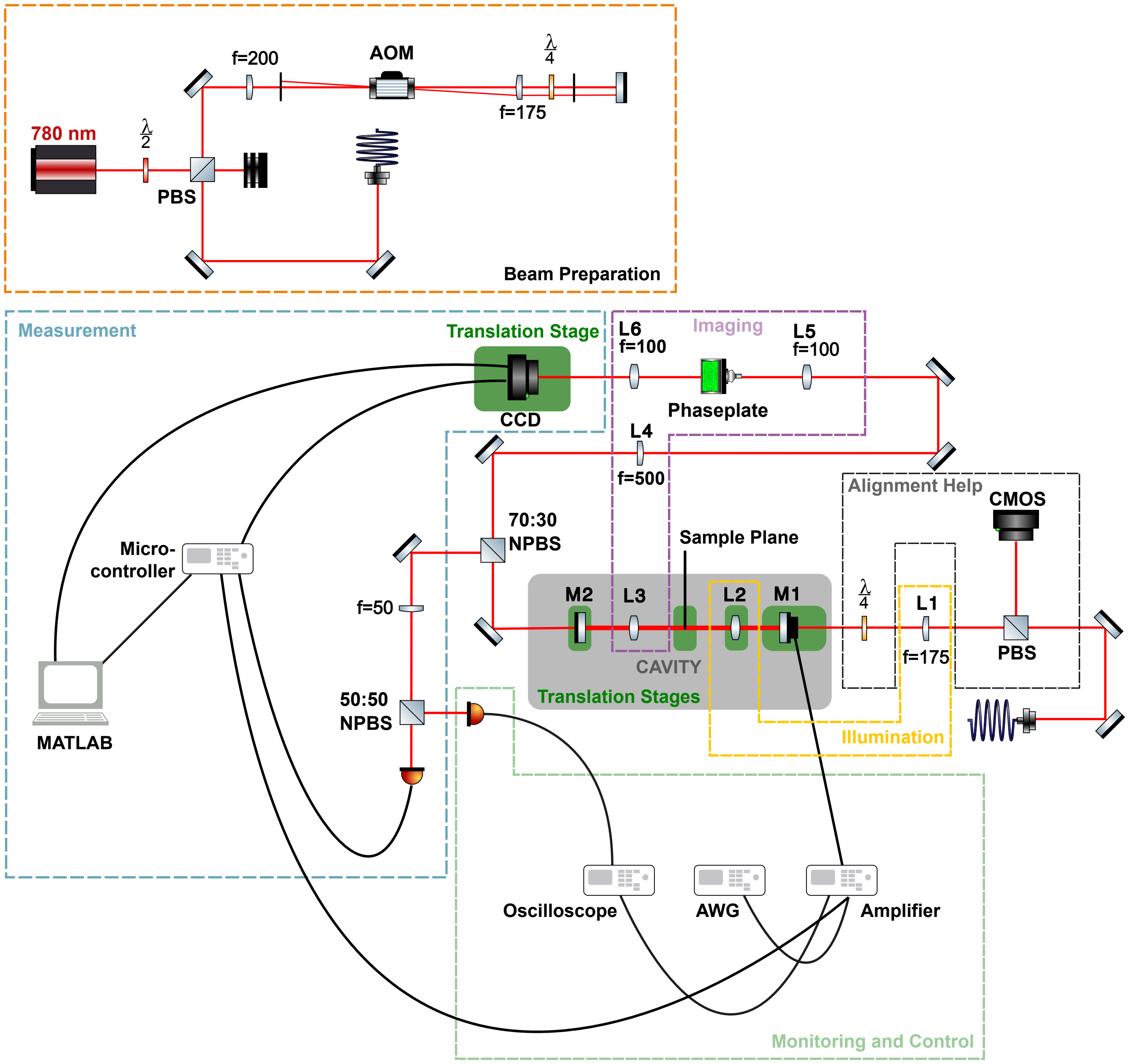

Supplementary Material Fig. 1. **Experimental Setup** A laser at 780 nm is coupled into a single-mode fiber following beam preparation, including power regulation and a double-pass acousto-optic modulator (AOM) setup for measuring the free spectral range (FSR) and potentially pulsing the laser. Subsequently, it encounters an alignment help, and a first lens focuses the beam on the in-coupling mirror, allowing for full-field illumination at the sample plane. Upon entering the cavity, the output is detected by two photodiodes and imaged by a camera after magnification, offering the possibility of implementing dark-field and phase-contrast imaging via a 4f setup. For real-time monitoring of the cavity response, the second photodiode is connected to an oscilloscope. To scan the cavity, the in-coupling mirror is affixed to a piezoelectric ring, enabling effective adjustment of the resonator's length.The focal lengths f are given in mm, the reflectivities R are given in %.

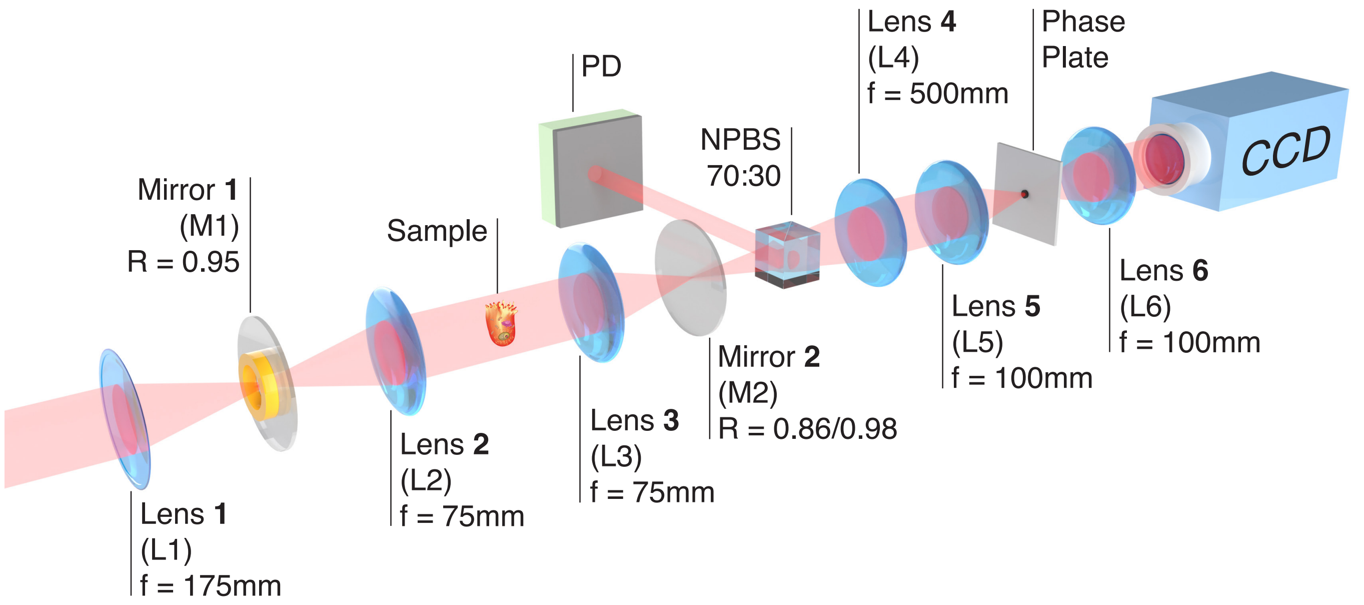

Supplementary Material Fig. 2. **Cavity setup** The self-imaging cavity is formed by two mirrors (M1 and M2) including lenses (L2 and L3) constituting a $4f$-setup. The cavity length is precisely controlled via a piezo ring mounted on M1. Light coupled into the resonator is focused on M1 to facilitate wide-field imaging in the sample plane, which is situated in the focal plane of lenses L2 and L3. After out-coupling, a non-polarizing beam splitter (NPBS) directs a fraction of the light to a photodiode for output monitoring. Lens 4 provides a magnification of 6.6, while an additional $4f$-setup enables various imaging techniques, including phase contrast. The resulting image of the sample is then detected by a CCD.

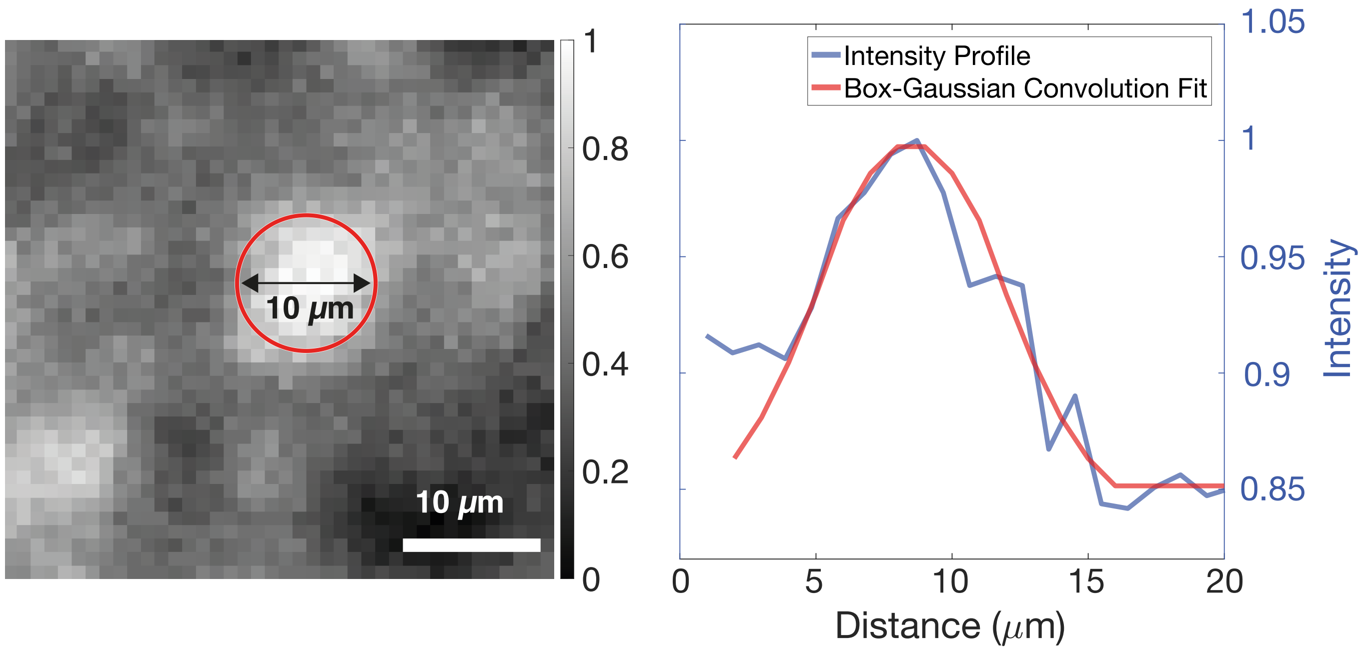

Supplementary Material Fig. 3. **Spot Size and Resolution Estimation** The left graphic shows the 10, μm hole when the cavity is on resonance. To assess the imaging capabilities of our setup, we aim to estimate the resolution. To do this, we fit a box function convolved with a Gaussian to the intensity profile of the hole, extracted from a single-pixel line cross-section. From this fit, we calculate the standard deviation, which serves as an estimate of the resolution. Theoretically, the lenses used can resolve features as small as approximately 2.8, μm.

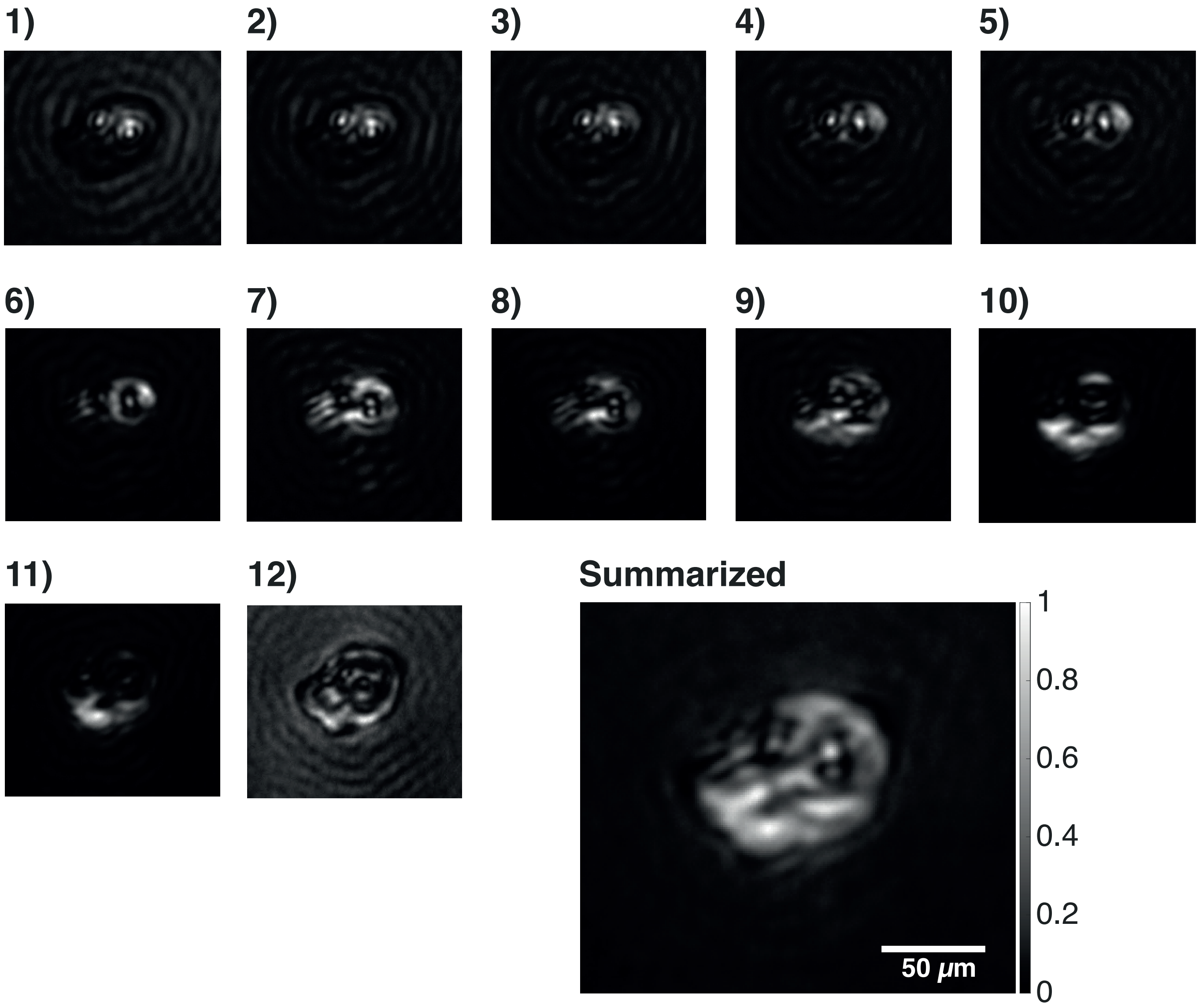

Supplementary Material Fig. 4. **Cheek Cell in self imaging Cavity** Micrsoscopy images for all cavity length in Fig. 3 of the main text. Images 1 through 12 depict various sections of a cheek cell as the cavity length is adjusted. These images were captured while the probe light was off resonance. Different phase shifts result in distinct resonance points for the light passing through the cell, effectively enabling sensitive measurements of the local optical path length through the sample. When these images are combined, they create a dark-field representation of the cell. Refining this technique could provide a method for measuring sample thickness and examining specific regions in detail.

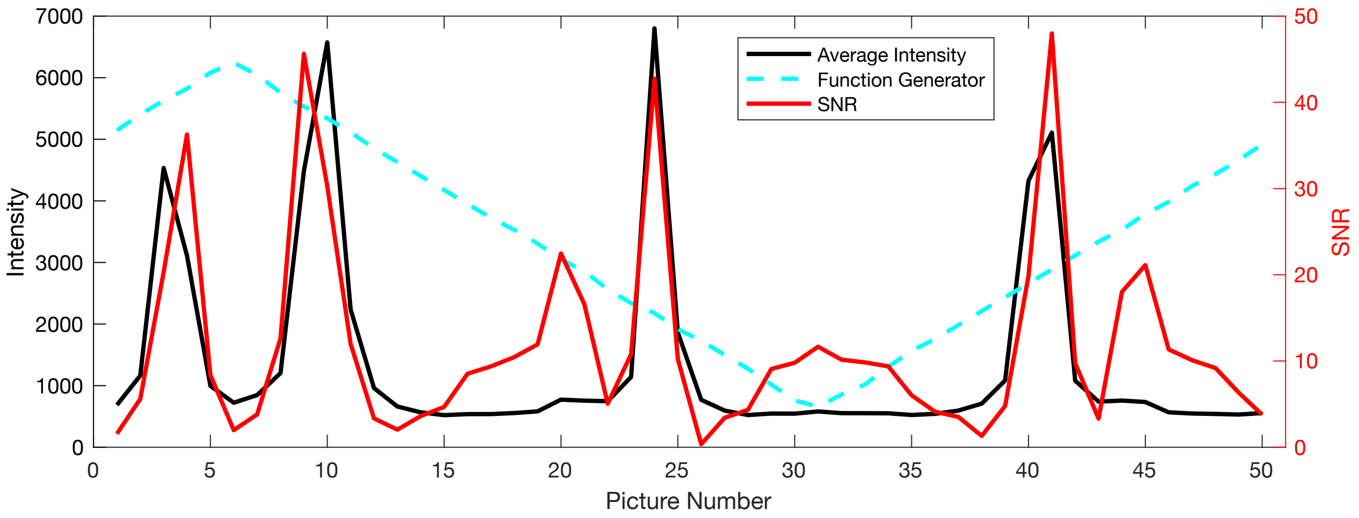

Supplementary Material Fig. 5. **Multiple scan across the resonance for cheek cell** Trace of the recorded transmitted intensity, the evaluated SNR and the piezo voltage (Function Generator) indicating the scan. One can clearly see that enhanced SNR on cavity resonance is a reproducible feature.
